# Association between optical coherence tomography–quantified retinal features and cardiovascular risk in cardiovascular–kidney–metabolic syndrome stages 0–3: An analysis of a prospective UK biobank cohort

**DOI:** 10.1371/journal.pone.0351945

**Published:** 2026-06-26

**Authors:** Chenhao Li, Sixiang Jia, Yang Yang, Qingru Zhu, Shudong Xia

**Affiliations:** Department of Cardiology, the Fourth Affiliated Hospital of School of Medicine, and International School of Medicine, Zhejiang University, Yiwu, China; The Chinese University of Hong Kong, HONG KONG

## Abstract

**Background:**

Cardiovascular–kidney–metabolic (CKM) syndrome is strongly associated with cardiovascular disease (CVD) and mortality. Stages 0–3 represent a key preclinical window for early intervention. Retinal optical coherence tomography (OCT) enables non-invasive evaluation of systemic microcirculation, but its prognostic value for CVD in CKM 0–3 remains undetermined.

**Methods:**

We conducted a prospective study of 40,516 UK Biobank participants with CKM syndrome stages 0–3 who had retinal OCT. Cox regression, trend tests, and predictive metrics (NRI, IDI, AUC) were used to evaluate associations and incremental prognostic value of retinal structural markers.

**Results:**

Baseline thinner retinal nerve fiber layer (RNFL) and macular thickness were independently associated with higher risks of incident CVD, coronary heart disease, all-cause mortality, and cardiovascular mortality over a subsequent median 14.6-year follow-up (all P < 0.001). Significant linear dose–response relationships were observed. Adding these markers to traditional risk models improved risk classification and discrimination (all P < 0.001).

**Conclusion:**

Thinner RNFL and macular thickness are independent predictors of adverse cardiovascular outcomes in CKM 0–3. Integrating these OCT-derived biomarkers improves risk stratification, supporting a non-invasive approach for early cardiovascular risk assessment in this high-risk population.

## Introduction

The American Heart Association (AHA) defined the Cardio-Kidney-Metabolic (CKM) syndrome in 2023 to highlight the mutual pathophysiological interactions among metabolic risk factors and chronic kidney disease (CKD) [1]. Mounting evidence indicates that the primary subsequent clinical burden of CKM syndrome is closely linked to cardiovascular diseases [2].The AHA classifies CKM syndrome into five stages, with stages 0–3 as preclinical; notably, approximately 90.8% of individuals meet the diagnostic criteria for these preclinical stages, with subclinical organ damage but no overt symptoms [3]. Accordingly, the AHA emphasizes prioritizing cardiovascular disease prevention in this population [4]. Microcirculatory dysfunction is a core pathophysiological mechanism underlying cardiovascular disease; thus, identifying early risk markers via convenient, non-invasive microcirculatory assessment tools is crucial for achieving precision prevention [5].

Retinal vessels share a common embryonic origin, anatomical structure, and physiological characteristics with renal and cardiac vessels [6,7], thus serving as a unique and accessible window to investigate the health and disease status of human microcirculation. As a classic tool for microcirculation observation, the value of fundus photographs in cardiovascular risk assessment has been validated by numerous studies [8,9]. In recent years, AI-based cardiovascular risk scoring models derived from fundus photographs have emerged [10]. However, fundus photograph interpretation is subject to inter-observer variability, and it is difficult to assess blood perfusion status and vascular permeability. Emerging evidence has confirmed that thinning of retinal neural layers precedes the onset of vascular morphological abnormalities [11].

Optical Coherence Tomography (OCT) enables non-invasive acquisition of ultra-high-resolution images and automatically segments and quantifies individual retinal layers (including the Retinal Nerve Fiber Layer (RNFL), Ganglion Cell-Inner Plexiform Layer (GC-IPL), etc.). These “OCT-derived retinal quantitative traits” have been shown to directly correlate with microvascular function [12,13], thus complementing fundus photographs. Emerging evidence indicates that RNFL thinning is associated with reduced high-density lipoprotein cholesterol (HDL-C) [14] and an increased risk of incident cardiovascular disease [15]. However, whether these associations can be extrapolated to the subclinical population with CKM syndrome stages 0–3, and whether other OCT-derived retinal quantitative traits (beyond RNFL) exhibit similar risk correlations, remains unclear. More importantly, no studies have explored the associations between OCT-derived retinal quantitative traits and cardiovascular or all-cause mortality in this population.

Therefore, the aim of this study was to use UK Biobank cohort data to prospectively investigate the association of OCT-derived retinal quantitative traits with the incidence of overall cardiovascular disease in individuals with CKM syndrome stages 0–3. Recognizing the central role of microvascular dysfunction across cardiovascular disease (CVD) yet prioritizing actionable clinical pathways, we conducted a prespecified, in-depth analysis on coronary heart disease(CHD) as the leading cause of morbidity and mortality in this population. We further examined associations with cardiovascular and all-cause mortality.

## Methods

### Study participants

Data for this study were derived from the UK Biobank, a large prospective community-based cohort. The project has been approved by the North West Multi-Centre Research Ethics Committee (MREC reference: 21/NW/0157) and conducted in compliance with the Declaration of Helsinki. Between 2006 and 2010, more than 500,000 participants were recruited. All participants provided written informed consent, completed touchscreen questionnaires, underwent physical examinations, and provided biological samples. After screening, a total of 80,240 participants underwent retinal OCT imaging. Detailed information on the overall study design and various assessment protocols is available on the official website (https://biobank.ndph.ox.ac.uk/ukb/index.cgi).

For the present study, anonymized data were accessed from the UK Biobank on 8 November 2025. No identifiable participant information was accessed during or after data collection for this analysis.

### Spectral-domain optical coherence tomography imaging protocol

Ocular assessment was introduced as an enhancement in 2009, applicable to 6 assessment centers across the United Kingdom [16]. Spectral-domain OCT imaging was performed using the Topcon 3D OCT 1000 Mk2 (Topcon Corp., Tokyo, Japan). Following assessments of visual acuity, autorefraction, and intraocular pressure (IOP), 3D macular volume scans were acquired under mesopic conditions without pupillary dilation (consistent scan settings: 512 A-scans per B-scan; 128 horizontal B-scans over a 6 × 6-mm area). All quantifiable retinal layer thickness measurements generated by the Topcon Advanced Boundary Segmentation (TABS) software (Version 1.6.1.1) were included in our analysis. The TABS segmentation algorithm was used to segment retinal layers within a 6-mm diameter circle centered at the true fovea, which was topographically defined across the macula by the Early Treatment Diabetic Retinopathy Study (ETDRS) grid [17]. The nine OCT-derived quantitative traits were: external limiting membrane-photoreceptor inner segment-outer segment complex (ELM-ISOS), inner nuclear layer (INL), retinal pigment epithelium (RPE), INL-ELM, INL-RPE, ISOS-RPE, ganglion cell-inner plexiform layer (GC-IPL), retinal nerve fiber layer (RNFL), and total macular thickness. Detailed methodological procedures have been described previously [18,19], and the reproducibility of the average thickness parameters derived from the TABS algorithm has been validated in prior studies, with intraclass correlation coefficients ranging from 0.933 to 0.954 [20]. The database field IDs corresponding to these traits and the quality control indicators used subsequently are provided in Supplementary S5 Table.

### Definition of outcomes

The primary outcomes of this study were the incidence of CVD, as well as cardiovascular and all-cause mortality, in participants with CKM syndrome stages 0–3. Participants were followed up from the date of initial assessment until the occurrence of the outcome or the end of follow-up (December 2, 2024). Outcome events were identified by linking to hospital admission records, primary care data, and national death registries. CVD was defined according to the International Classification of Diseases, 10th Revision (ICD-10) (see Supplementary S4 Table for details). Mortality data were obtained by linking to the National Health Service (NHS) Death Registry, based on the underlying cause of death.

### Definition of CKM syndrome across stages 0–3

Consistent with previous studies [21], CKM syndrome stages 0–3 is defined as follows:

*Stage 0: The absence of risk factors for CKM syndrome, defined as normal Body mass index (BMI), Waist circumference (WC), blood glucose, systolic blood pressure (SBP), diastolic blood pressure (DBP), lipid levels, renal function, and no evidence of CVD.*

*Stage 1: Characterized by the presence of at least one of the following:*

*(1) BMI ≥ 25 kg/m²; (2) WC ≥ 88 cm in women or≥102 cm in men; and (3) prediabetes, defined as 5.7% ≤ glycated hemoglobin (HbA1c)≤6.4%.*

*Stage 2: Presence of moderate to high-risk CKD, defined as an estimated Glomerular Filtration Rate (eGFR) between 30 and 60 mL/min/1.73 m², and at least one of the following metabolic risk factors: (1) hypertension; (2) diabetes; (3) triglycerides(TG)≥135 mg/dL; and (4) diagnosis of metabolic syndrome, which is defined as meeting at least three of the following criteria: increased WC, reduced HDL-C (<40 mg/dL in men or<50 mg/dL in women), TG > 150 mg/dL, elevated BP (SBP ≥ 130 mmHg or DBP ≥ 80 mmHg), and prediabetes.*

*Stage 3: Individuals exhibiting subclinical CVD, as indicated by a high predicted CVD risk (>10 points) based on the Systematic Coronary Risk Evaluation 2 (SCORE2) model, or those with advanced-stage CKD, indicated by a 15 < eGFR < 30 mL/min/1.73 m². The SCORE2 model is a validated risk prediction algorithm developed for European populations to estimate the risk of adverse cardiovascular events in individuals without diagnosed CVD [* 22 *].*

Detailed information on the classification of CKM syndrome stages 0–3 is shown in S1 Table.

### Covariates

Based on previous studies [23,24], we identified several baseline variables as potential covariates. These included age (continuous, in years), sex, ethnicity (White vs. non-White), Townsend Deprivation Index (TDI; continuous, higher values indicate greater deprivation), educational level (university degree or higher vs. others), employment status (employed vs. unemployed), smoking status (Never, Ever, Current), alcohol consumption frequency (ranging from never to daily or almost daily), and sleep duration (<7 hours/day, 7–8 hours/day, > 8 hours/day). Also included were blood pressure (SBP and DBP, continuous, in mmHg), fasting plasma glucose (FPG; continuous, in mmol/L), HDL-C, and low-density lipoprotein cholesterol (LDL-C; continuous, in mmol/L). Given the extremely low missing rate of covariates, participants with missing covariate data were excluded in the primary analysis. Detailed information on covariate definitions and missing data statistics is provided in S2–S3 Tables.

### Inclusion and exclusion criteria

**Fig 1:**
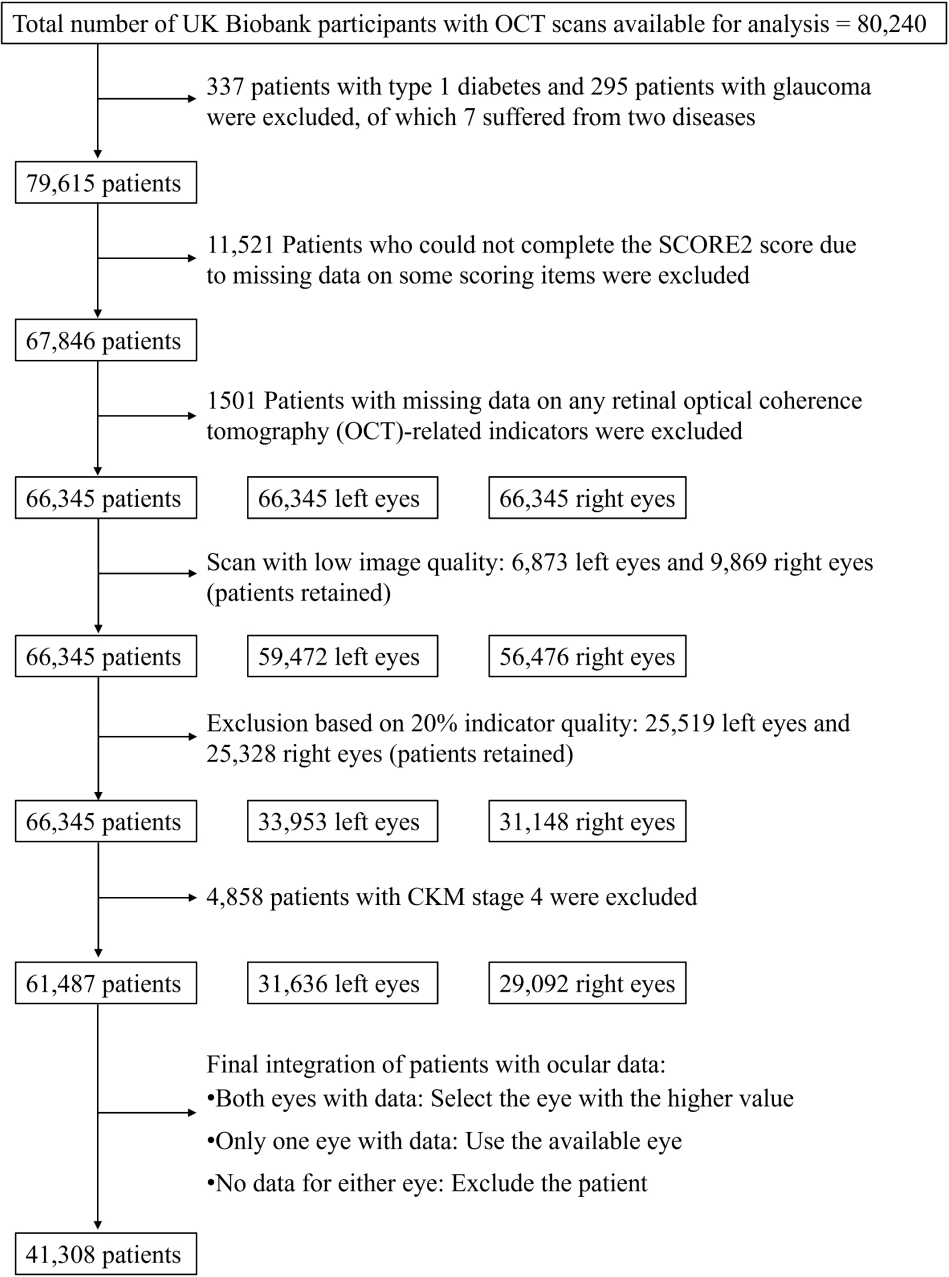
Flowchart of selecting study population in the UK Biobank.

This study used 80,240 participants with OCT scan data from the UK Biobank as the initial sample, subsequently excluding the following individuals: patients with type 1 diabetes or glaucoma, those unable to complete the SCORE2 score, participants with missing OCT-related indicator data, patients with CKM stage 4, and those with missing usable data for both eyes after ocular data integration. Additionally, quality control (QC) was performed on ocular data, with QC dimensions including image quality score, internal limiting membrane (ILM) metrics, validity count, and motion metrics. Based on this, participants with suboptimal OCT image quality (image quality <45 points or other quality metrics in the lowest 20th percentile) were excluded [18,19]. A total of 41,308 participants were finally included in this study, with the detailed screening process shown in Fig 1 after further excluding participants with missing covariates, 40,516 individuals with CKM syndrome stages 0–3 were included in the final analysis.

### Statistical analysis

Descriptive statistics were used to summarize participants’ baseline characteristics, stratified by CVD status. Continuous variables were presented as mean ± standard deviation (SD) or median (interquartile range, IQR) based on their distribution, while categorical variables were expressed as frequencies (percentages). Between-group comparisons used independent samples t-test (normally distributed continuous variables), Mann-Whitney U test (non-normally distributed), and Pearson’s chi-squared test (categorical variables).

Cox proportional hazards models, a widely used method for survival analysis, were used to examine the associations and dose-response relationships between OCT-derived retinal quantitative traits and the four clinical outcomes. The proportional hazards (PH) assumption was evaluated using Schoenfeld residuals for each model. Prior to analysis, extreme values were removed by excluding observations outside the 1st to 99th percentiles for each OCT trait to minimize bias from outliers. In multivariable-adjusted Cox models, hazard ratios (HRs) and 95% confidence intervals (CIs) were estimated per 1-standard deviation increment in each OCT trait. To address multiple testing, the Benjamini–Hochberg false discovery rate (FDR) procedure was applied to the 9 OCT traits for each outcome to control for type I error inflation. Statistical significance was defined as FDR-adjusted P < 0.05 for all analyses. OCT traits that showed significant associations with all four outcomes (incident CVD, incident CHD, all-cause mortality, and cardiovascular mortality) at FDR P ≤ 0.05 were retained for subsequent stratification and predictive performance analyses. To clarify the strength and pattern of these associations, the retained OCT traits were categorized into quartiles (with Q1 as the reference group), and HRs with 95% CIs were calculated for Q2–Q4; the incidence rate per 1000 person-years was also reported. Finally, to further clarify the dose-response relationships, Kaplan-Meier (KM) cumulative risk curves were constructed for OCT traits with significant multivariable associations. The log-rank test was used to evaluate overall differences across quartile groups. Quartile categories were converted to ordinal variables for trend tests in multivariable Cox models to assess linear associations; simultaneously, quadratic terms were included in the adjusted Cox models to evaluate potential non-linearity, with significance determined by the P-value of the quadratic term.

Furthermore, two comparative models were constructed to evaluate the incremental prognostic value of OCT traits: the basic model included age, sex, ethnicity, SBP, DBP, smoking status, alcohol status, sleep status, employment status, TDI, FPG, BMI, eGFR, HDL-C and LDL-C; the new model added the screened OCT-derived retinal quantitative traits to the base model. To compare the predictive performance and discriminative ability of the two models, the net reclassification improvement (NRI), integrated discrimination improvement (IDI), and area under the receiver operating characteristic curve (AUC) were calculated.

Sensitivity analyses were conducted to verify the robustness of the results: ① Repeating the association analyses after excluding participants with outcome events within the first 2 years of follow-up to reduce potential reverse causality; ② Repeating the analyses after imputing missing covariates using multiple imputation by chained equations (MICE); ③ To assess competing risk events, competing risk models were used to re-evaluate the associations; ④ Repeating the analyses using data from the thinner eye (instead of the thicker eye in the primary analysis) for participants with complete bilateral data; ⑤ Repeating the analyses including all samples (without excluding extreme values) to test the impact of outlier exclusion. Additionally, stratified subgroup analyses were performed by age (<60 vs. ≥ 60 years), sex (female vs. male), ethnicity (White vs. non-White), and CKM stages (0–1 vs. 2–3) to explore the consistency of associations.

All statistical analyses were performed using R software (Version 4.5.2) and Python (Version 3.10), with statistical significance set at two-tailed P < 0.05.

## Results

### Baseline characteristics of the study population

After excluding participants with missing covariates, a total of 40,516 individuals with CKM syndrome stages 0–3 were included (median age [IQR]: 57.0 [50.0, 63.0] years; 53.4% female). Their associations with the overall incidence of cardiovascular disease (CVD) are summarized in Table 1. Compared with participants who did not develop CVD, those with incident CVD were elder, had a higher proportion of males, lower educational levels, a higher proportion of White individuals, were current smokers, reported higher alcohol consumption frequency, insufficient sleep duration, and were in CKM syndrome stage 3. Additionally, individuals with incident CVD had higher baseline levels of SBP, DBP and FPG (all P < 0.001). Among OCT-derived retinal quantitative traits, except for INL thickness (P = 0.195), the thicknesses of GC-IPL, RNFL, ELM-ISOS, ISOS-RPE, and overall macular thickness were significantly thinner in individuals with incident CVD than in those without (all P < 0.001); RPE, INL-ELM, and INL-RPE thicknesses also showed statistically significant differences (all P < 0.05).

**Table 1:** Baseline characteristics of study population stratified by the incidence of overall CVD.

	Total population	CVD incidence		P value
		Yes	No	
N	40,516	7,339	33,177	
Age (years)	57.00 (50.00-63.00)	62.00 (56.00-66.00)	56.00 (49.00-62.00)	<0.001
Sex				
Female	21635 (53.4%)	3083 (42.0%)	18552 (55.9%)	<0.001
male	18881 (46.6%)	4256 (58.0%)	14625 (44.1%)	<0.001
Ethnicity				
White	37531 (92.6%)	6930 (94.4%)	30601 (92.2%)	<0.001
Non-White	2985 (7.4%)	409 (5.6%)	2576 (7.8%)	<0.001
Education				
College or university degree	15083 (37.2%)	2244 (30.6%)	12839 (38.7%)	<0.001
Others	25433 (62.8%)	5095 (69.4%)	20338 (61.3%)	<0.001
Employment				
Empolyed	39674 (97.9%)	7195 (98.0%)	32479 (97.9%)	0.468
Unempolyed	842 (2.1%)	144 (2.0%)	698 (2.1%)	0.468
Smoking				
Never smoker	22835 (56.4%)	3595 (49.0%)	19240 (58.0%)	<0.001
Ever smoker	13959 (34.5%)	2923 (39.8%)	11036 (33.3%)	<0.001
Current smoker	3722 (9.2%)	821 (11.2%)	2901 (8.7%)	<0.001
Alcohol consumption				
Never drinking	3019 (7.5%)	631 (8.6%)	2388 (7.2%)	<0.001
Special occasions only	4660 (11.5%)	923 (12.6%)	3737 (11.3%)	<0.001
One to three time a month	4680 (11.6%)	824 (11.2%)	3856 (11.6%)	<0.001
Oner or twice a week	10183 (25.1%)	1712 (23.3%)	8471 (25.5%)	<0.001
Three or four times a week	9485 (23.4%)	1586 (21.6%)	7899 (23.8%)	<0.001
Daily or almost daily	8489 (21.0%)	1663 (22.7%)	6826 (20.6%)	<0.001
CKM stage				
0	9814 (24.2%)	1104 (15.0%)	8710 (26.3%)	<0.001
1	10518 (26.0%)	1761 (24.0%)	8757 (26.4%)	<0.001
2	18392 (45.4%)	3726 (50.8%)	14666 (44.2%)	<0.001
3	1792 (4.4%)	748 (10.2%)	1044 (3.1%)	<0.001
Sleep duration				
<7 hours/day	10002 (24.7%)	2015 (27.5%)	7987 (24.1%)	<0.001
7-8 hours/day	27767 (68.5%)	4685 (63.8%)	23082 (69.6%)	<0.001
>8 hours/day	2747 (6.8%)	639 (8.7%)	2108 (6.4%)	<0.001
Townsend	−1.83 (−3.45-0.75)	−1.87 (−3.48-0.80)	−1.82 (−3.45-0.73)	0.912
SBP (mmHg)	135.50 (123.50-148.50)	141.50 (129.00-154.00)	134.00 (123.00-147.00)	<0.001
DBP (mmHg)	81.50 (75.00-88.00)	83.00 (76.50-89.50)	81.00 (74.50-88.00)	<0.001
HDL-C (mmol/L)	1.45 (1.21-1.73)	1.38 (1.15-1.64)	1.46 (1.23-1.74)	<0.001
LDL-C (mmol/L)	3.54 (3.00-4.12)	3.50 (2.92-4.12)	3.55 (3.01-4.12)	<0.001
FPG(mmol/L)	4.99 (4.70-5.32)	5.04 (4.73-5.39)	4.98 (4.69-5.30)	<0.001
GC-IPL (μm)	74.22 (70.12-78.14)	73.45 (69.25-77.44)	74.41 (70.33-78.29)	<0.001
RNFL (μm)	29.27 (26.46-32.50)	28.61 (25.81-31.91)	29.43 (26.60-32.64)	<0.001
INL (μm)	32.61 (31.10-34.19)	32.57 (31.07-34.15)	32.62 (31.11-34.20)	0.195
RPE (μm)	24.92 (23.37-27.05)	24.86 (23.39-26.79)	24.93 (23.36-27.12)	0.002
Macular (μm)	278.45 (269.67-287.24)	276.90 (267.97-285.82)	278.78 (270.05-287.52)	<0.001
ELM-ISOS (μm)	23.48 (22.71-24.38)	23.30 (22.51-24.17)	23.53 (22.74-24.42)	<0.001
INL-ELM (μm)	80.78 (76.63-85.03)	80.96 (76.75-85.24)	80.75 (76.60-84.99)	0.023
INL-RPE (μm)	142.64 (137.56-147.82)	142.35 (137.49-147.60)	142.70 (137.57-147.87)	0.011
ISOS-RPE (μm)	38.89 (36.36-41.17)	38.58 (36.27-40.85)	38.97 (36.38-41.23)	<0.001

Data are presented as mean (SD) or median (interquartile range) for continuous variables and n (%) for categorical variables. P values: t-test, Mann-Whitney U, or chi-squared test as appropriate; two-tailed.

### Associations between OCT-derived retinal quantitative traits and outcome events

After multivariable adjustment and Benjamini–Hochberg false discovery rate (FDR) correction, only two OCT-derived retinal quantitative traits, retinal nerve fiber layer (RNFL) thickness and overall macular thickness, were significantly associated with all four endpoints. These endpoints included all-cause mortality, cardiovascular mortality, incident cardiovascular CVD, and incident CHD) Both traits showed significant inverse associations with all outcomes (Table 2).

**Table 2:** Associations between OCT-derived retinal quantitative traits and clinical outcomes: Multivariate Cox proportional hazards model analyses.

Exposure	Outcome	HR (95%CI)	Raw P	FDR P
ELM_ISOS	All-cause mortality	0.97 (0.93-1.01)	0.156	0.213
	Cardiovascular mortality	0.90 (0.82-1.00)	0.040	0.083
	Cardiovascular incidence	0.96 (0.94-0.98)	<0.001	0.001
	CHD incidence	0.95 (0.92-0.99)	0.013	0.038
GCIPL	All-cause mortality	0.92 (0.89-0.96)	<0.001	<0.001
	Cardiovascular mortality	0.88 (0.80-0.96)	0.006	0.018
	Cardiovascular incidence	0.97 (0.94-0.99)	0.004	0.007
	CHD incidence	0.98 (0.94-1.01)	0.196	0.352
INL	All-cause mortality	1.00 (0.96-1.04)	0.977	0.977
	Cardiovascular mortality	0.96 (0.88-1.05)	0.410	0.461
	Cardiovascular incidence	1.00 (0.98-1.02)	0.893	0.893
	CHD incidence	1.00 (0.96-1.04)	0.996	0.996
INL_ELM	All-cause mortality	0.97 (0.94-1.01)	0.190	0.213
	Cardiovascular mortality	0.94 (0.86-1.03)	0.221	0.285
	Cardiovascular incidence	1.00 (0.97-1.02)	0.846	0.893
	CHD incidence	1.00 (0.96-1.04)	0.924	0.996
INL_RPE	All-cause mortality	0.96 (0.93-1.00)	0.060	0.108
	Cardiovascular mortality	0.91 (0.83-1.00)	0.046	0.083
	Cardiovascular incidence	0.98 (0.95-1.00)	0.048	0.072
	CHD incidence	0.98 (0.95-1.02)	0.372	0.558
ISOS_RPE	All-cause mortality	0.94 (0.90-0.97)	0.001	0.002
	Cardiovascular mortality	0.92 (0.84-1.01)	0.081	0.121
	Cardiovascular incidence	0.95 (0.92-0.97)	<0.001	<0.001
	CHD incidence	0.96 (0.92-1.00)	0.035	0.078
Macular_Thickness	All-cause mortality	0.92 (0.88-0.95)	<0.001	<0.001
	Cardiovascular mortality	0.83 (0.76-0.92)	<0.001	0.001
	Cardiovascular incidence	0.94 (0.92−0.97)	<0.001	<0.001
	CHD incidence	0.95 (0.92-0.99)	0.009	0.038
RNFL	All-cause mortality	0.92 (0.89-0.96)	<0.001	<0.001
	Cardiovascular mortality	0.85 (0.77-0.93)	<0.001	0.003
	Cardiovascular incidence	0.92 (0.90-0.94)	<0.001	<0.001
	CHD incidence	0.91 (0.88-0.95)	<0.001	<0.001
RPE	All-cause mortality	0.97 (0.93-1.01)	0.187	0.213
	Cardiovascular mortality	0.97 (0.87-1.07)	0.488	0.488
	Cardiovascular incidence	1.01 (0.98-1.03)	0.627	0.806
	CHD incidence	1.01 (0.97-1.05)	0.658	0.846

These two significant retinal measures were further categorized into quartiles to explore risks of clinical outcomes according to quartiles of significant OCT-derived retinal quantitative traits. Graded inverse associations and clear linear dose-response relationships were observed across quartiles. The incidence rates of all clinical outcomes per 1000 person-years decreased progressively from Q1 to Q4.

For overall macular thickness: Each 1-SD increase had a HR of 0.92 (95% CI: 0.88–0.95) for all-cause mortality, 0.83 (95% CI: 0.76–0.92) for cardiovascular mortality, 0.94 (95% CI: 0.92–0.97) for incident CVD, and 0.95 (95% CI: 0.92–0.99) for incident CHD; compared with the lowest quartile (Q1), multivariate-adjusted HRs for the highest quartile (Q4) were 0.83 (95% CI: 0.74–0.92), 0.58 (95% CI: 0.43–0.77), 0.88 (95% CI: 0.82–0.94), and 0.86 (95% CI: 0.77–0.95) for the above outcomes, respectively (Table 3).

**Table 3:** Risks of clinical outcomes according to quartiles of significant OCT-derived retinal quantitative traits.

Exposures	All-cause mortality		Cardiovascular mortality		Overall CVD Incidence		CHD Incidence	
	Incidence rate	HR(95%CI)	Incidence rate	HR(95%CI)	Incidence rate	HR(95%CI)	Incidence rate	HR(95%CI)
RNFL								
Per SD increment		0.92 (0.89-0.96)		0.85 (0.77-0.93)		0.92 (0.9-0.94)		0.91 (0.88-0.95)
Quartile Q1	6.12	Reference	1.16	Reference	17.03	Reference	6.67	Reference
Quartile Q2	5.19	0.98 (0.89-1.09)	0.94	0.96 (0.77-1.21)	14.3	0.94 (0.89-1.0)	5.42	0.92 (0.83-1.02)
Quartile Q3	4.38	0.89 (0.8-0.99)	0.67	0.75 (0.58-0.97)	12.75	0.87 (0.82-0.93)	4.95	0.88 (0.8-0.98)
Quartile Q4	3.91	0.82 (0.73-0.91)	0.59	0.68 (0.52-0.89)	12.23	0.82 (0.77-0.87)	4.51	0.79 (0.72-0.88)
Macular								
Per SD increment		0.92 (0.88-0.95)		0.83 (0.76-0.92)		0.94 (0.92-0.97)		0.95 (0.92-0.99)
Quartile Q1	6.26	Reference	1.18	Reference	16.79	Reference	6.58	Reference
Quartile Q2	5.07	0.9 (0.81-1.0)	0.93	0.89 (0.71-1.13)	14.4	0.92 (0.86-0.98)	5.24	0.86 (0.78-0.95)
Quartile Q3	4.53	0.87 (0.78-0.96)	0.84	0.88 (0.69-1.12)	13.35	0.9 (0.85-0.96)	5.26	0.91 (0.82-1.01)
Quartile Q4	3.9	0.83 (0.74-0.92)	0.5	0.58 (0.43-0.77)	12.11	0.88 (0.82-0.94)	4.63	0.86 (0.77-0.95)

Models were adjusted for age, TDI, FPG, HDL-C, LDL-C, systolic over diastolic blood pressure, sex, ethnicity, smoking status, alcohol consumption, educational level, sleep duration, and employment status.

Note: incidence rates were shown as per 1000 person-years.

For RNFL thickness: Each 1-SD increase had a HR of 0.92 (95% CI: 0.89–0.96) for all-cause mortality, 0.85 (95% CI: 0.77–0.93) for cardiovascular mortality, 0.92 (95% CI: 0.90–0.94) for incident CVD, and 0.91 (95% CI: 0.88–0.95) for incident CHD; compared with the lowest quartile (Q1), multivariate-adjusted HRs for the highest quartile (Q4) were 0.82 (95% CI: 0.73–0.91), 0.68 (95% CI: 0.52–0.89), 0.82 (95% CI: 0.77–0.87), and 0.79 (95% CI: 0.72–0.88) for the above outcomes, respectively (Table 3).

**Fig 2:**
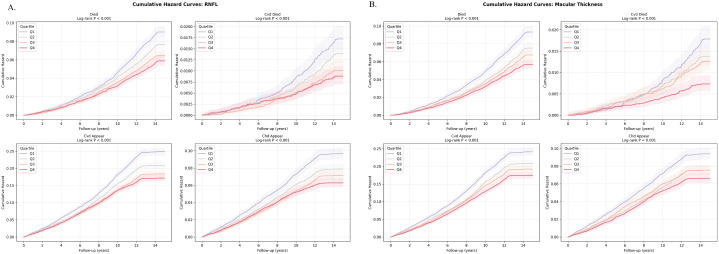
Kaplan-Meier cumulative risk curves for four clinical outcomes (incident CVD, CHD, all-cause mortality, cardiovascular mortality) stratified by quartiles of RNFL thickness (A) and macular thickness (B). Q1 = thinnest, Q4 = thickest. Log-rank P values are shown.

In the dose-response relationship refinement phase, Kaplan-Meier (KM) cumulative risk curves were plotted by quartile groups for overall macular thickness and RNFL thickness—both confirmed to have significant associations in multivariate analyses. The curves showed no crossings, with log-rank test P-values < 0.001 (Fig 2), indicating significant differences in cumulative outcome incidences between quartile groups. To quantify linearity, quartile groups were converted to ordinal variables for trend tests in multivariate Cox models. Results showed per SD-based and quartile-based trend test P-values < 0.001 for both traits (Table 4), confirming significant linear associations with the four outcomes. Concurrently, quadratic terms were constructed using standardized OCT traits to fit Cox models incorporating linear terms, quadratic terms, and all covariates for non-linear assessment. All quadratic term P-values across outcomes were > 0.05, with HRs (95% CIs) including 1.0 (Table 4), indicating no significant non-linear associations and further validating the robustness of linear dose-response relationships.

**Table 4:** Linear trend tests and quadratic term analyses for the associations between OCT-derived retinal quantitative traits and clinical outcomes.

Exposure	P-trend (Per SD-based)	P-trend (Quartile-based)	Quadratic_Coefficient	Quadratic_HR（95%CI）	Quadratic_P_value
Macular					
All-cause mortality	<0.001	<0.001	0.0219	1.0221(0.9926-1.0525)	0.1429
CHD incidence	0.0106	0.007	0.0053	1.0053(0.9769-1.0345)	0.7171
Cardiovascular incidence	<0.001	<0.001	0.0122	1.0123(0.9941-1.0308)	0.1874
Cardiovascular mortality	<0.001	<0.001	−0.0246	0.9757(0.9053-1.0516)	0.5199
RNFL					
All-cause mortality	<0.001	<0.001	0.0042	1.0042(0.9752-1.0341)	0.7771
CHD incidence	<0.001	<0.001	0.0154	1.0155(0.9875-1.0443)	0.2815
Cardiovascular incidence	<0.001	<0.001	0.0046	1.0046(0.9867-1.0228)	0.6156
Cardiovascular mortality	<0.001	<0.001	−0.0098	0.9903(0.9195-1.0666)	0.7965

Models were adjusted for age, TDI, FPG, HDL-C, LDL-C, systolic over diastolic blood pressure, sex, ethnicity, smoking status, alcohol consumption, educational level, sleep duration, and employment status.

### Incremental prognostic value of OCT-derived retinal quantitative traits

RNFL thickness and overall macular thickness were separately added to the base model to evaluate their ability to improve the predictive performance for CVD and mortality in patients with CKM syndrome stages 0–3. As summarized in Table 5, after incorporating these two traits into the base model, although there were no significant differences in the AUC, the NRI and IDI for all outcomes were significantly enhanced—with RNFL demonstrating superior overall incremental prognostic value (NRI: all-cause mortality = 0.1307, cardiovascular mortality = 0.1857, incident CVD = 0.09, incident CHD = 0.0712; IDI: all-cause mortality = 0.0277, cardiovascular mortality = 0.1458, incident CVD = 0.0169, incident CHD = 0.0231; all P < 0.001).

**Table 5:** Incremental predictive value of RNFL and overall macular thickness for cardiovascular disease and mortality in participants with CKM syndrome stages 0–3.

Exposures	Continuous NRI (95% CI)	P value	IDI (95% CI)	P value	AUC (95% CI)	P value
Coronary heart disease incidence						
Basic model	Ref	–	Ref	–	0.7524 (0.7453-0.7598)	Ref
Basic model + Macular Thickness	0.0262 (−0.0006-0.053)	0.056	0.0043 (0.0001-0.0084)	0.045	0.7467 (0.7376-0.7562)	0.347
Basic model + RNFL Thickness	0.0712 (0.0446-0.0979)	<0.001	0.0231 (0.0136-0.0325)	<0.001	0.743 (0.7335-0.7517)	0.111
Cardiovascular incidence						
Basic model	Ref	–	Ref	–	0.7399 (0.7349-0.7455)	Ref
Basic model + Macular Thickness	0.0376 (0.0195-0.0557)	<0.001	0.0056 (0.003-0.0082)	<0.001	0.7349 (0.7286-0.7416)	0.241
Basic model + RNFL Thickness	0.09 (0.072-0.108)	<0.001	0.0169 (0.0125-0.0214)	<0.001	0.7309 (0.7242-0.7377)	0.042
Cardiovascular mortality						
Basic model	Ref	–	Ref	–	0.7986 (0.7841-0.8128)	Ref
Basic model + Macular Thickness	0.1157 (0.0521-0.1794)	<0.001	0.1412 (0.0528-0.2296)	0.002	0.8039 (0.7867-0.8227)	0.652
Basic model + RNFL Thickness	0.1857 (0.1232-0.2481)	<0.001	0.1458 (0.0566-0.235)	0.001	0.8017 (0.7829-0.8191)	0.793
All-cause mortality						
Basic model	Ref	–	Ref	–	0.7621 (0.7548-0.7692)	Ref
Basic model + Macular Thickness	0.0405 (0.0129-0.0681)	0.004	0.0254 (0.0137-0.0371)	<0.001	0.7596 (0.7509-0.769)	0.672
Basic model + RNFL Thickness	0.1307 (0.1036-0.1579)	<0.001	0.0277 (0.0163-0.0391)	<0.001	0.7575 (0.7486-0.7664)	0.436

The basic model included variables for age, sex, ethnicity, SBP, DBP, smoking status, alcohol status, sleep status, employment status, TDI, FPG, BMI, eGFR, HDL-C and LDL-C.

### Sensitivity and subgroup analyses

We verified the robustness of our results via multiple sensitivity and stratified subgroup analyses, with core findings highly consistent with the primary analysis (S1–S9 Figs). In sensitivity analyses, the associations between traits and outcomes remained essentially unchanged whether excluding early follow-up outcomes to mitigate reverse causality, handling missing data via MICE, adjusting for competing outcome events via competing risk models, modifying eye data selection rules (using the thinner eye), or including outlier samples. Additionally, after stratification by age, sex, ethnicity, and CKM stages, association trends in all subgroups were consistent with the overall results—with stronger associations observed in males, individuals aged ≥60 years, White participants, and those with CKM stages 2–3.

## Discussion

Utilizing the large prospective UK Biobank cohort, this study focused on participants with CKM syndrome stages 0–3 to explore the associations between OCT-derived retinal quantitative traits and CVD-related outcomes as well as mortality. Following multidimensional screening and validation, we finally identified RNFL thickness and overall macular thickness as core associated traits, which can effectively enhance the incremental predictive value for the aforementioned outcomes, and RNFL exhibits a more significant association strength.

Our findings align closely with previous research. Chen Y et al. [15] have confirmed that OCT-quantified RNFL thinning is positively associated with overall cardiac disease burden, a result validated in a Chinese diabetic cohort. Building on this, our study further expands the evidence base in three key aspects: First, we not only refined the focus from “overall cardiac disease” to specific outcomes (incident CVD and CHD) but also, for the first time, verified its independent associations with all-cause and cardiovascular mortality in participants with CKM syndrome stages 0–3. Notably, these mortality associations were stronger than those for incident CVD, highlighting RNFL’s prognostic value across the full outcome chain from onset to death. Second, compared to the tertile grouping used in that study, we adopted more granular quartile grouping (Q1–Q4) and systematically validated the robustness of linear associations between RNFL and all outcomes via linear trend tests and quadratic term nonlinearity tests, ruling out nonlinear inflection points. Third, we conducted head-to-head comparisons of multiple OCT-derived retinal quantitative traits. Results showed overall macular thickness also had significant independent associations with cardiovascular outcomes, with predictive trends consistent with RNFL. Importantly, RNFL exhibited the strongest predictive performance among these traits, and holds promise as a potential quantitative adjunct beyond traditional risk factors in clinical cardiovascular risk assessment.

Consistent with these findings, cross-sectional studies also report thinner RNFL in patients with chronic heart failure or CHD compared to healthy individuals [25,26]; this association has been further confirmed in multi‑ethnic Asian populations [27]. The precise pathophysiological mechanism linking RNFL and macular thinning to cardiovascular disease remains incompletely understood, though current evidence suggests it may involve reduced retinal blood flow [28]. In individuals with CKM syndrome stages 0–3, metabolic abnormalities such as insulin resistance and dyslipidemia not only promote cardiovascular disease [21,24] but also contribute to systemic microcirculatory dysfunction [29]. Prior studies indicate that sublingual microcirculatory impairment correlates with vascular complications in CKM syndrome and could serve as a potential staging tool [30]. At the retinal level, against the background of CKM‑related systemic microcirculatory disturbance, retinal vessels may undergo compensatory constriction to prioritize perfusion of vital organs (e.g., heart and brain), thereby reducing inner retinal blood supply and increasing susceptibility to ischemic injury [6]. Thus, such microangiopathy is likely a key driver of the observed reductions in RNFL and macular thickness [31]. Future studies incorporating OCT angiography to directly measure retinal blood flow will help validate this proposed pathophysiological pathway.

Surveys have shown that public enthusiasm for ophthalmic screening is significantly higher than that for cardiovascular disease screening [32]. Additionally, patients with CKM syndrome often have comorbidities such as diabetes and hypertension, who already require routine fundus examinations. Thus, integrating cardiovascular disease risk assessment into ophthalmic screening not only aligns with the medical-seeking behaviors of the target population but also boasts strong clinical feasibility. Furthermore, due to the speed and non-invasiveness of OCT technology, it is more acceptable to patients and suitable for large-scale screening compared to traditional invasive tests. A hierarchical assessment workflow is recommended in clinical practice. When thinning of the RNFL or macular thickness is detected, primary ocular diseases such as glaucoma should be excluded via specialized ophthalmic examinations. A comprehensive assessment integrating smoking, alcohol consumption and other cardiovascular risk factors as well as patients’ symptoms is then performed, and high-risk individuals will be timely referred for cardiovascular examinations to achieve early intervention and prevention. During patient counseling, it should be stated clearly that reduced RNFL thickness is a non-specific finding, which can also be observed in patients with schizophrenia and various neurodegenerative diseases. Although relevant studies suggest that such retinal alterations are mostly secondary to hypertension, diabetes mellitus and other cardiovascular comorbidities [33], targeted screening is still necessary for those with corresponding symptoms. Meanwhile, clinicians need to elaborate on the relationship among RNFL changes, microcirculation and cardiovascular health, so that patients can understand the predictive implication of this marker and actively cooperate with follow-up and treatment.

This study has notable strengths including a large sample size, prospective design, and long-term follow-up. Moreover, multiple sensitivity and stratified analyses have further enhanced the robustness and credibility of the findings. Nevertheless, several limitations should be acknowledged: First, despite previous confirmation of the reproducibility of risk factor analyses in the UK Biobank [34], the inherent “healthy volunteer” bias of this cohort may introduce selection bias [35]. Second, although potential confounders were adjusted for, residual confounding and reverse causation bias may still exist in observational studies, which limits the validity of causal inferences to a certain extent. For instance, the observed J-shaped association between alcohol consumption and CVD (never drinkers having higher rates than moderate drinkers) and the higher CVD rates in long sleepers (>8 hours/day) are likely driven by such biases (e.g., healthy volunteer bias, reverse causation) rather than causal effects. However, this potential bias has been mitigated through sensitivity analysis excluding outcomes occurring within the first two years of follow-up. Third, the study population is predominantly White, which limits the generalizability of the results. Due to the relatively small sample size of non-White ethnic groups, subgroup analyses showed weaker associations between retinal structural parameters and cardiovascular outcomes in this population. Therefore, further validation in cohorts with more diverse ethnic compositions is warranted. Fourth, this study only used baseline OCT measurements and did not conduct longitudinal assessment of dynamic changes in retinal structural parameters, which provides a direction for future research. Fifth, although this study excluded glaucoma, a well-recognized confounder, it did not rule out other ocular diseases that may affect retinal structure, such as age-related macular degeneration. This constitutes an important limitation of the study. Considering that the diagnostic codes for other ocular diseases in the UK Biobank are mainly derived from inpatient records, there is a risk of under-ascertainment. Therefore, excluding cases solely based on such codes may introduce misclassification bias. Future studies incorporating standardized systematic eye examinations are thus warranted.

## Conclusion

Following a series of screenings of OCT-derived retinal quantitative traits, we identified RNFL and overall macular thickness as independently significant correlates of CVD onset and mortality risk in patients with CKM syndrome stages 0–3. Their thickness was significantly negatively associated with adverse outcome risk, with thinner thickness correlating with higher risk. Incremental predictive analyses demonstrated that incorporating these two traits into traditional risk prediction models yielded statistically significant improvements in NRI and IDI, indicating their value as potential predictors of cardiovascular risk in this subclinical population.

## Supporting information

S1 Table Detailed definition of cardiovascular-kidney-metabolic syndrome. (DOCX)

S2 Table Measures of covariates at baseline in the UK Biobank. (DOCX)

S3 Table Variance Inflation Factors (VIF) and missing data characteristics of covariates in participants with CKM syndrome stages 0–3. (DOCX)

S4 Table Definitions and Corresponding ICD Codes for Outcome Events. (DOCX)

S5 Table OCT-Derived Retinal Quantitative Features and Quality Control Fields in UK Biobank. Abbreviations: ELM = External Limiting Membrane; GCIPL = Ganglion Cell-Inner Plexiform Layer; INL = Inner Nuclear Layer; ISOS = Inner Segment Outer Segment; ILM = Internal Limiting Membrane; RPE = Retinal Pigment Epithelium; RNFL = Retinal Nerve Fibre Layer.
(DOCX)

S1 Fig Subgroup analysis stratified by age (<60 vs ≥ 60 years) for the associations between RNFL and overall macular thickness and cardiovascular-related outcomes in CKM stages 0–3. Subgroup analysis stratified by age (<60 vs ≥ 60 years) for the associations between RNFL and overall macular thickness and cardiovascular-related outcomes in CKM stages 0–3. Data were presented as hazard ratio and 95% confidence interval. Models were adjusted for Townsend deprivation index, fasting plasma glucose, high-density lipoprotein cholesterol (HDL), low-density lipoprotein cholesterol (LDL), systolic over diastolic blood pressure, sex, ethnicity, smoking status, alcohol consumption, educational level, sleep duration, and employment status. *Abbreviations*: CVD, cardiovascular disease; CHD, coronary heart disease.
(DOCX)

S2 Fig Subgroup analysis stratified by sex (female vs male) for the associations between RNFL and overall macular thickness and cardiovascular-related outcomes in CKM stages 0–3. Subgroup analysis stratified by sex (female vs male) for the associations between RNFL and overall macular thickness and cardiovascular-related outcomes in CKM stages 0–3 Data were presented as hazard ratio and 95% confidence interval. Models were adjusted for age, Townsend deprivation index, fasting plasma glucose, high-density lipoprotein cholesterol (HDL), low-density lipoprotein cholesterol (LDL), systolic over diastolic blood pressure, ethnicity, smoking status, alcohol consumption, educational level, sleep duration, and employment status. *Abbreviations*: CVD, cardiovascular disease; CHD, coronary heart disease.
(DOCX)

S3 Fig Subgroup analysis stratified by CKM stage (0–1 vs 2–3) for the associations between RNFL and overall macular thickness and cardiovascular-related outcomes. Subgroup analysis stratified by CKM stage (0–1 vs 2–3) for the associations between RNFL and overall macular thickness and cardiovascular-related outcomes. Data were presented as hazard ratio and 95% confidence interval. Models were adjusted for age, Townsend deprivation index, fasting plasma glucose, high-density lipoprotein cholesterol (HDL), low-density lipoprotein cholesterol (LDL), systolic over diastolic blood pressure, sex, ethnicity, smoking status, alcohol consumption, educational level, sleep duration, and employment status. *Abbreviations*: CVD, cardiovascular disease; CHD, coronary heart disease.
(DOCX)

S4 Fig Subgroup analysis stratified by ethnicity (Whites vs non-Whites) for the associations between RNFL and overall macular thickness and cardiovascular-related outcomes in CKM stages 0–3. Subgroup analysis stratified by ethnicity (Whites vs non-Whites) for the associations between RNFL and overall macular thickness and cardiovascular-related outcomes in CKM stages 0–3. Data were presented as hazard ratio and 95% confidence interval. Models were adjusted for age, Townsend deprivation index, fasting plasma glucose, high-density lipoprotein cholesterol (HDL), low-density lipoprotein cholesterol (LDL), systolic over diastolic blood pressure, sex, smoking status, alcohol consumption, educational level, sleep duration, and employment status. *Abbreviations*: CVD, cardiovascular disease; CHD, coronary heart disease.
(DOCX)

S5 Fig Sensitivity analysis for the associations between RNFL and overall macular thickness and cardiovascular-related outcomes in CKM stages 0–3: excluding early (≤2 years) outcomes. Sensitivity analysis for the associations between RNFL and overall macular thickness and cardiovascular-related outcomes in CKM stages 0–3: excluding early (≤2 years) outcomes. Data were presented as hazard ratio and 95% confidence interval. Models were adjusted for age, Townsend deprivation index, fasting plasma glucose, high-density lipoprotein cholesterol (HDL), low-density lipoprotein cholesterol (LDL), systolic over diastolic blood pressure, sex, smoking status, alcohol consumption, educational level, sleep duration, and employment status. *Abbreviations*: CVD, cardiovascular disease; CHD, coronary heart disease.
(DOCX)

S6 Fig Sensitivity analysis for the associations between RNFL and overall macular thickness and cardiovascular-related outcomes in CKM stages 0–3: multiple imputation for missing covariates. Sensitivity analysis for the associations between RNFL and overall macular thickness and cardiovascular-related outcomes in CKM stages 0–3: multiple imputation for missing covariates. Data were presented as hazard ratio and 95% confidence interval. Models were adjusted for age, Townsend deprivation index, fasting plasma glucose, high-density lipoprotein cholesterol (HDL), low-density lipoprotein cholesterol (LDL), systolic over diastolic blood pressure, sex, smoking status, alcohol consumption, educational level, sleep duration, and employment status. *Abbreviations*: CVD, cardiovascular disease; CHD, coronary heart disease.
(DOCX)

S7 Fig Sensitivity analysis for the associations between RNFL and overall macular thickness and cardiovascular-related outcomes in CKM stages 0–3: including all samples without excluding extreme values (1st and 99th percentiles). Sensitivity analysis for the associations between RNFL and overall macular thickness and cardiovascular-related outcomes in CKM stages 0–3: including all samples without excluding extreme values (1st and 99th percentiles). Data were presented as hazard ratio and 95% confidence interval. Models were adjusted for age, Townsend deprivation index, fasting plasma glucose, high-density lipoprotein cholesterol (HDL), low-density lipoprotein cholesterol (LDL), systolic over diastolic blood pressure, sex, smoking status, alcohol consumption, educational level, sleep duration, and employment status. *Abbreviations*: CVD, cardiovascular disease; CHD, coronary heart disease.
(DOCX)

S8 Fig Sensitivity analysis for the associations between RNFL and overall macular thickness and cardiovascular-related outcomes in CKM stages 0–3: using thinner eye data instead of thicker eye data for bilateral complete cases. Sensitivity analysis for the associations between RNFL and overall macular thickness and cardiovascular-related outcomes in CKM stages 0–3: using thinner eye data instead of thicker eye data for bilateral complete cases. Data were presented as hazard ratio and 95% confidence interval. Models were adjusted for age, Townsend deprivation index, fasting plasma glucose, high-density lipoprotein cholesterol (HDL), low-density lipoprotein cholesterol (LDL), systolic over diastolic blood pressure, sex, smoking status, alcohol consumption, educational level, sleep duration, and employment status. *Abbreviations*: CVD, cardiovascular disease; CHD, coronary heart disease.
(DOCX)

S9 Fig Sensitivity analysis for the associations between RNFL and overall macular thickness and cardiovascular and coronary outcomes in CKM stages 0–3: competing risk models. Sensitivity analysis for the associations between RNFL and overall macular thickness and cardiovascular and coronary outcomes in CKM stages 0–3: competing risk models. Data were presented as hazard ratio and 95% confidence interval. Models were adjusted for age, Townsend deprivation index, fasting plasma glucose, high-density lipoprotein cholesterol (HDL), low-density lipoprotein cholesterol (LDL), systolic over diastolic blood pressure, sex, smoking status, alcohol consumption, educational level, sleep duration, and employment status. *Abbreviations*: CVD, cardiovascular disease; CHD, coronary heart disease.
(DOCX)

## Abbreviations

AUC: Area under the curve
BMI: Body mass index
CHD: Coronary heart disease
CKD: Chronic kidney disease
CKM: Cardiovascular–kidney–metabolic
CI: Confidence interval
CVD: Cardiovascular disease
DBP: Diastolic blood pressure
ELM: External Limiting Membrane
eGFR: Estimated Glomerular Filtration Rate
FDR: False Discovery Rate
FPG: Fasting plasma glucose
GC-IPL: Ganglion Cell-Inner Plexiform Layer
HbA1c: Glycated hemoglobin
HDL-C: High-density lipoprotein cholesterol
HR: Hazard ratio
HNS: National Health Service
ICD-10: International Classification of Diseases, Tenth Revision
ILM: Internal Limiting Membrane
INL: Inner Nuclear Layer
IOP: Intraocular pressure
ISOS: Inner Segment Outer Segment
IQR: Interquartile ranges
KM: Kaplan–Meier
LDL-C: Low-density lipoprotein cholesterol
MACE: Major Adverse Cardiovascular Events
MICE: Multiple imputation by chained equations
MREC: Multi-Centre Research Ethics Committee
NRI: Net reclassification index
OCT: Optical Coherence Tomography
PH: Proportional Hazards
QC: Quality control
Q1: Quartile 1
RPE: Retinal Pigment Epithelium
RNFL: Retinal Nerve Fiber Layer
SBP: Systolic blood pressure
SD: Standard deviation
SCORE2: Systematic Coronary Risk Evaluation 2
TABS: Topcon Advanced Boundary Segmentation
TDI: Townsend Deprivation Index
TG: Triglyceride
WC: Waist circumference
