# Supplementary material for: Association between optical coherence tomography–quantified retinal features and cardiovascular risk in cardiovascular–kidney–metabolic syndrome stages 0–3: An analysis of a prospective UK biobank cohort
Source: PLoS One. 2026 Jun 26;21(6):e0351945. doi: 10.1371/journal.pone.0351945 (PMC13308834; doi:10.1371/journal.pone.0351945)
Supplement: S1 Table — (DOCX) [file pone.0351945.s001.docx]

**Table S1.** Detailed definition of cardiovascular-kidney-metabolic syndrome

| **Stages** | **Definition** |
| --- | --- |
| Stage 0 | Stage 0 is defined as the absence of risk factors for cardiovascular-kidney-metabolic (CKM) syndrome, including normal body mass index (BMI), waist circumference (WC), blood glucose, systolic blood pressure (SBP), diastolic blood pressure (DBP), lipid levels, renal function, and no evidence of cardiovascular disease (CVD). |
| Stage 1 | Stage 1 was characterized by the existence of at least one of the following metabolic dysfunctions: (1) BMI ≥25 kg/m^2^; (2) WC ≥88/102 cm in women/men; (3) prediabetes: 5.7%≤ glycated hemoglobin (HbA1c) ≤6.4%. |
| Stage 2 | Stage 2 is defined as the presence of moderate to high-risk chronic kidney disease (CKD) and the existence of one or more of the following metabolic risk factors: (1) hypertension; (2) diabetes; (3) triglycerides (TG) ≥ 135 mg/dL; (4) diagnosis of metabolic syndrome.  The moderate to high-risk CKD was defined as an estimated Glomerular Filtration Rate (eGFR) between 30 and 60 mL/min/1.73 m^2^, and eGFR was calculated using the CKD-EPI 2021 creatinine-based equation proposed by the Chronic Kidney Epidemiology Collaboration.  Hypertension was defied as a self-reported history of hypertension, and/or SBP ≥130 mmHg, and/or DBP ≥80 mmHg, and/or the intake of anti-hypertensive medications.  Diabetes was defined as HbA1c ≥6.4%, and/or a diagnostic history of diabetes before baseline, and/or the intake of glucose-lowering medications.  Metabolic syndrome is defined as meeting at least three of the following criteria: (1) increased WC; (2) reduced high-density lipoprotein cholesterol (HDL-C < 40 mg/dL in men or < 50 mg/dL in women); (3) high TG (TG > 150 mg/dL); (4) elevated BP (SBP ≥ 130 mmHg or DBP ≥ 80 mmHg); or (5) prediabetes. |
| Stage 3 | Stage 3 involves individuals with subclinical CVD or advanced-stage CKD.  Participants with subclinical CVD was defined as a high predicted CVD risk based on the Systematic Coronary Risk Evaluation 2 (SCORE2) model.  Participants with advanced-stage CKD include those with an eGFR < 30 mL/min/1.73 m^2^. |
