# Supplementary material for: Association between optical coherence tomography–quantified retinal features and cardiovascular risk in cardiovascular–kidney–metabolic syndrome stages 0–3: An analysis of a prospective UK biobank cohort
Source: PLoS One. 2026 Jun 26;21(6):e0351945. doi: 10.1371/journal.pone.0351945 (PMC13308834; doi:10.1371/journal.pone.0351945)
Supplement: S2 Table — (DOCX) [file pone.0351945.s002.docx]

| **Table S2.** Measures of covariates at baseline in the UK Biobank | | | | |
| --- | --- | --- | --- | --- |
| **Variable** | **Filed ID** | **Question or description** | **Categories from raw data** | **Categories for the current study** |
| Ethnicity | 21000 | Ethnic background | White;  Mixed;  Asian or Asian British;  Black or Black British;  Chinese;  Other ethnic group. | 0 = White;  1 = Non-White |
| Education | 6138 | Qualifications | College or University degree;  A levels/AS levels or equivalent;  O levels/GCSEs or equivalent;  CSEs or equivalent;  NVQ or HND or HNC or equivalent;  Other professional qualifications eg: nursing, teaching. | 0 = College or university degree;  1 = Others. |
| Employment | 6142 | Current employment status | In paid employment or self-employed;  Retired;  Looking after home and/or family;  Unable to work because of sickness or disability;  Unemployed;  Doing unpaid or voluntary work;  Full or part-time student;  None of the above. | 0 = Employed (including those paid employment or self-employed, retired, doing unpaid or voluntary work, or being full or part time students);  1 = Unemployed. |
| Townsend deprivation index | 22189 | Townsend deprivation index at recruitment | Continuous. | A higher value indicates greater deprivation. |
| Smoking | 20116 | The current/past smoking status of the participant. | Never; Past; Current. | 0 = Never smoker;  1 = Ever smoker;  2= Current smoker. |
| Alcohol consumption | 1558 | About how often do you drink alcohol? | Never; Special occasions only; One to three times a month; Once or twice a week; Three or four times a week; Daily or almost daily. | 1 = Never drinking;  2 = Special occasions only;  3 = One to three time a month;  4 = Oner or twice a week;  5 = Three or four times a week;  6 = Daily or almost daily. |
| Sleep duration | 1160 | About how many hours sleep do you get in every 24 hours? (please include naps)? | Continuous. | 0 = <7 hours/day;  1 = 7-8 hours/day;  2= >8 hours/day. |
