# Supplementary material for: Association between optical coherence tomography–quantified retinal features and cardiovascular risk in cardiovascular–kidney–metabolic syndrome stages 0–3: An analysis of a prospective UK biobank cohort
Source: PLoS One. 2026 Jun 26;21(6):e0351945. doi: 10.1371/journal.pone.0351945 (PMC13308834; doi:10.1371/journal.pone.0351945)
Supplement: S3 Table — (DOCX) [file pone.0351945.s003.docx]

**Table S3** Variance Inflation Factors (VIF) and missing data characteristics of covariates in participants with CKM syndrome stages 0–3

| Covariate | VIF | Missing Count | Missing Ratio(%) |
| --- | --- | --- | --- |
| age | 1.25 | 0 | 0 |
| Townsend deprivation index | 1.11 | 57 | 0.09 |
| fasting plasma glucose | 1.02 | 83 | 0.13 |
| HDL-C | 1.35 | 0 | 0 |
| LDL-C | 1.02 | 124 | 0.2 |
| systolic over diastolic | 1.21 | 0 | 0 |
| sex | 1.32 | 0 | 0 |
| Ethnicity | 1.13 | 0 | 0 |
| Smoking | 1.06 | 0 | 0 |
| Alcohol consumption | 1.23 | 42 | 0.07 |
| Education | 1.04 | 0 | 0 |
| Sleep duration | 1.01 | 276 | 0.44 |
| Employment | 1.02 | 640 | 1.02 |
