# Supplementary material for: Association between optical coherence tomography–quantified retinal features and cardiovascular risk in cardiovascular–kidney–metabolic syndrome stages 0–3: An analysis of a prospective UK biobank cohort
Source: PLoS One. 2026 Jun 26;21(6):e0351945. doi: 10.1371/journal.pone.0351945 (PMC13308834; doi:10.1371/journal.pone.0351945)
Supplement: S4 Table — (DOCX) [file pone.0351945.s004.docx]

**Table S4** Definitions and Corresponding ICD Codes for Outcome Events

| Outcome Events | ICD-10 |
| --- | --- |
| Cardiovascular Disease | Rheumatic Heart Diseases (I05-I09) |
|  | Hypertensive Heart and Renal Disease (I11-I13) |
|  | Ischemic Heart Diseases (I20-I25) |
|  | Acute Pulmonary Thromboembolism (I26) |
|  | Non-Rheumatic Valvular, Endocardial and Myocardial Diseases (I33-I38) |
|  | Cardiomyopathies (I42) |
|  | Cardiac Arrhythmias (I44-I49) |
|  | Heart Failure (I50) |
|  | Cerebrovascular Diseases (I60-I69) |
|  | Diseases of Arteries, Arterioles and Capillaries (Including TIA) (I70-I74) |
|  | Venous Thrombotic Diseases (I80-I82) |
| Coronary Heart Disease | Ischemic Heart Diseases (I20-I25) |
