# Supplementary material for: Association between optical coherence tomography–quantified retinal features and cardiovascular risk in cardiovascular–kidney–metabolic syndrome stages 0–3: An analysis of a prospective UK biobank cohort
Source: PLoS One. 2026 Jun 26;21(6):e0351945. doi: 10.1371/journal.pone.0351945 (PMC13308834; doi:10.1371/journal.pone.0351945)
Supplement: S5 Table — Abbreviations: ELM = External Limiting Membrane; GCIPL = Ganglion Cell-Inner Plexiform Layer; INL = Inner Nuclear Layer; ISOS = Inner Segment Outer Segment; ILM = Internal Limiting Membrane; RPE = Retinal Pigment Epithelium; RNFL = Retinal Nerve Fibre Layer. (DOCX) [file pone.0351945.s005.docx]

**Table S5.** OCT-Derived Retinal Quantitative Features and Quality Control Fields in UK Biobank

| Indicator | **Field ID (Left Eye)** | **Field ID (Right Eye)** |
| --- | --- | --- |
| Average ELM-ISOS thickness | 28520 | 28521 |
| Average INL-ELM thickness | 28512 | 28513 |
| Average INL-RPE thickness | 28536 | 28537 |
| Average ISOS-RPE thickness | 28528 | 28529 |
| Average GCIPL layer thickness | 28504 | 28505 |
| Average INL thickness | 28502 | 28503 |
| Average RNFL thickness | 28500 | 28501 |
| Overall average RPE thickness | 27822 | 27823 |
| Overall macular thickness | 27800 | 27801 |
| ILM indicator | 28542 | 28543 |
| Image quality | 28552 | 28553 |
| Max motion delta | 28548 | 28549 |
| Max motion factor | 28550 | 28551 |
| Min motion correlation | 28546 | 28547 |
| Valid count | 28544 | 28545 |

*Abbreviations:*

ELM = External Limiting Membrane; GCIPL = Ganglion Cell-Inner Plexiform Layer; INL = Inner Nuclear Layer; ISOS = Inner Segment Outer Segment; ILM = Internal Limiting Membrane; RPE = Retinal Pigment Epithelium; RNFL = Retinal Nerve Fibre Layer.
