# Supplementary material for: Association between optical coherence tomography–quantified retinal features and cardiovascular risk in cardiovascular–kidney–metabolic syndrome stages 0–3: An analysis of a prospective UK biobank cohort
Source: PLoS One. 2026 Jun 26;21(6):e0351945. doi: 10.1371/journal.pone.0351945 (PMC13308834; doi:10.1371/journal.pone.0351945)
Supplement: S4 Fig — Subgroup analysis stratified by ethnicity (Whites vs non-Whites) for the associations between RNFL and overall macular thickness and cardiovascular-related outcomes in CKM stages 0–3. Data were presented as hazard ratio and 95% confidence interval. Models were adjusted for age, Townsend deprivation index, fasting plasma glucose, high-density lipoprotein cholesterol (HDL), low-density lipoprotein cholesterol (LDL), systolic over diastolic blood pressure, sex, smoking status, alcohol consumption, educational level, sleep duration, and employment status. Abbreviations: CVD, cardiovascular disease; CHD, coronary heart disease. (DOCX) [file pone.0351945.s009.docx]

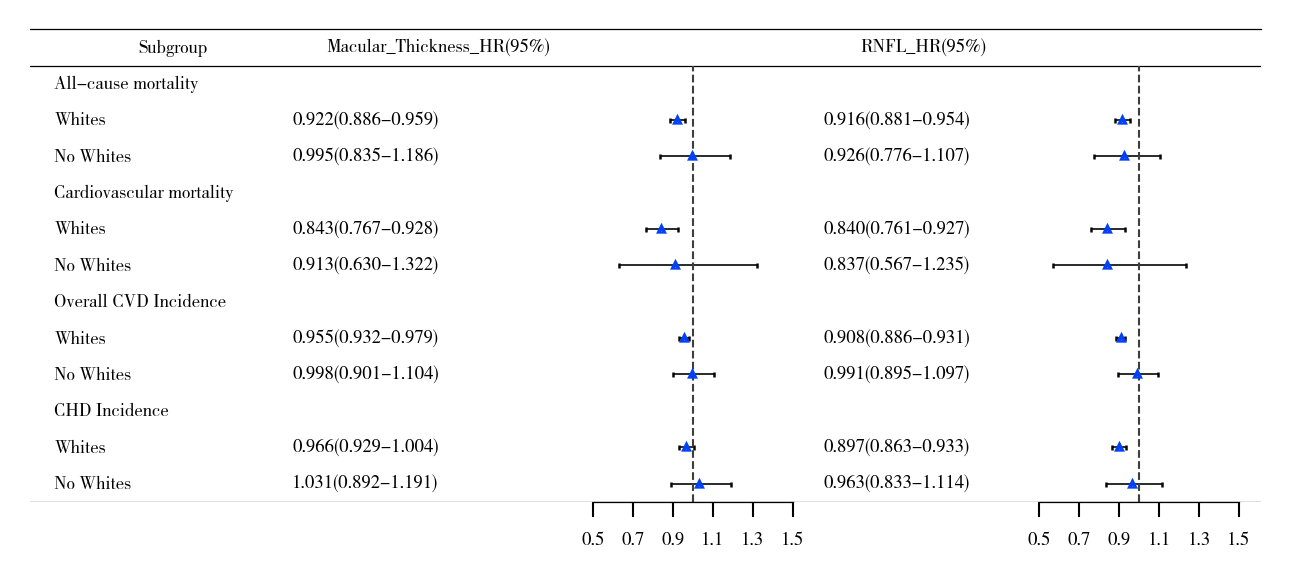


**Figure S4.** Subgroup analysis stratified by ethnicity (Whites vs non-Whites) for the associations between RNFL and overall macular thickness and cardiovascular-related outcomes in CKM stages 0-3

Data were presented as hazard ratio and 95% confidence interval. Models were adjusted for age, Townsend deprivation index, fasting plasma glucose, high-density lipoprotein cholesterol (HDL), low-density lipoprotein cholesterol (LDL), systolic over diastolic blood pressure, sex, smoking status, alcohol consumption, educational level, sleep duration, and employment status.

*Abbreviations*: CVD, cardiovascular disease; CHD, coronary heart disease;
