# Supplementary material for: Association between optical coherence tomography–quantified retinal features and cardiovascular risk in cardiovascular–kidney–metabolic syndrome stages 0–3: An analysis of a prospective UK biobank cohort
Source: PLoS One. 2026 Jun 26;21(6):e0351945. doi: 10.1371/journal.pone.0351945 (PMC13308834; doi:10.1371/journal.pone.0351945)
Supplement: S9 Fig — Sensitivity analysis for the associations between RNFL and overall macular thickness and cardiovascular and coronary outcomes in CKM stages 0–3: competing risk models. Data were presented as hazard ratio and 95% confidence interval. Models were adjusted for age, Townsend deprivation index, fasting plasma glucose, high-density lipoprotein cholesterol (HDL), low-density lipoprotein cholesterol (LDL), systolic over diastolic blood pressure, sex, smoking status, alcohol consumption, educational level, sleep duration, and employment status. Abbreviations: CVD, cardiovascular disease; CHD, coronary heart disease. (DOCX) [file pone.0351945.s014.docx]

|  | Cardiovascular mortality | | | Overall CVD Incidence | | | CHD Incidence | | |
| --- | --- | --- | --- | --- | --- | --- | --- | --- | --- |
|  | Incidence_Rate | HR（95%CI） | P_value | Incidence_Rate | HR（95%CI） | P_value | Incidence_Rate | HR（95%CI） | P_value |
| Macular_Thickness | |  |  |  |  |  |  |  |  |
| Per SD increment | | 0.84 (0.77-0.92) | <0.001 |  | 0.95 (0.92-0.97) | <0.001 |  | 0.96 (0.92-0.99) | 0.018 |
| Quartile | 1.18 | Reference |  | 16.79 | Reference |  | 6.58 | Reference |  |
| Quartile | 0.93 | 0.91 (0.72-1.15) | 0.41 | 14.4 | 0.93 (0.87-0.99) | 0.017 | 5.24 | 0.87 (0.78-0.96) | 0.005 |
| Quartile | 0.84 | 0.89 (0.70-1.14) | 0.36 | 13.35 | 0.91 (0.85-0.97) | 0.003 | 5.26 | 0.92 (0.83-1.02) | 0.098 |
| Quartile | 0.5 | 0.58 (0.44-0.78) | <0.001 | 12.11 | 0.88 (0.83-0.95) | <0.001 | 4.63 | 0.87 (0.78-0.96) | 0.008 |
| RNFL |  |  |  |  |  |  |  |  |  |
| Per SD increment | | 0.84 (0.77-0.93) | <0.001 |  | 0.92 (0.90-0.94) | <0.001 |  | 0.91 (0.88-0.95) | <0.001 |
| Quartile | 1.16 | Reference |  | 17.03 | Reference |  | 6.67 | Reference |  |
| Quartile | 0.94 | 0.96 (0.76-1.20) | 0.7 | 14.3 | 0.94 (0.88-1.00) | 0.051 | 5.42 | 0.92 (0.83-1.01) | 0.086 |
| Quartile | 0.67 | 0.75 (0.58-0.96) | 0.024 | 12.75 | 0.87 (0.82-0.93) | <0.001 | 4.95 | 0.89 (0.80-0.98) | 0.019 |
| Quartile | 0.59 | 0.67 (0.51-0.88) | 0.004 | 12.23 | 0.82 (0.76-0.87) | <0.001 | 4.51 | 0.79 (0.71-0.88) | <0.001 |

**Figure S9.** Sensitivity analysis for the associations between RNFL and overall macular thickness and cardiovascular and coronary outcomes in CKM stages 0-3: competing risk models

Data were presented as hazard ratio and 95% confidence interval. Models were adjusted for age, Townsend deprivation index, fasting plasma glucose, high-density lipoprotein cholesterol (HDL), low-density lipoprotein cholesterol (LDL), systolic over diastolic blood pressure, sex, smoking status, alcohol consumption, educational level, sleep duration, and employment status.

*Abbreviations*: CVD, cardiovascular disease; CHD, coronary heart disease;
